# Glycerine

**Published:** 1856-06

**Authors:** H. Wardner


### THE NORTH-WESTERN

### MEDICAL AND SURGICAL JOURNAL.

(NEW SERIES.)

VOL. V. JUNE, 1856. NO. 6.

### ORIGINAL COMMUNICATIONS.

ARTICLE I.—*Glycerine. Inaugural Thesis presented to the Faculty of Rush Medical College, Feb. 1st*, 1856. By H. WARDNER.

Glycerine (*glukus*—sweet) is the base of all fats and oils that are saponafiable. It was discovered by Scheele, in 1789, in the process of lead-plaster making, and was called by him the sweet principle of oils. Twenty years afterwards, Chevreul found it to be the base of fats and fat oils. It consists of one equivalent of the hypothetical radical glycerule ($C^6 H^7$,) five equivalents of oxygen ($O^5$,) and one of water ($H^0$,) and in chemical language, is termed the hydrated oxide of glycerule.

It is obtained in an impure state during soap making, which fact has given it the popular name of "soapmaker's waste." From this source, hundreds of tons are annually thrown away.

A second source, is in the manufacture of the common lead-plaster, *i. e.*, by heating together a suitable oil, protoxide of lead, and water. An insoluble soap of lead is formed, while the glycerine remains in solution with the water.

The solution is then treated with sulphuretted hydrogen, to free it from any of the lead which might be remaining; then digested with animal charcoal, to purify it from the gaseous impurities which might be present. If the oil used is vegetable, it frees the solution from any of the coloring and odoriferous principles; and if it be animal, it frees it from any tainted animal odor. The solution is then filtered, and lastly evaporated *in vacuo*, at the temperature of the atmosphere. This is the source from which it has mostly been obtained.

A third source, is in the stearine candle manufacture, where the glycerine is separated by the lime saponafication. Mr. G. F. Wilson states, that many of the purifiers of glycerine seem to have preferred this source.

A fourth process for obtaining it, is given by Mr. G. F. Wilson, in an article on "Glycerine and its Uses," presented before the British Association, at Glasgow, in September, 1855, which is as follows: "Steam, at a temperature of 550° to 600° Fahr., is introduced into a distillatory apparatus, containing a quantity of palm oil. The fatty acids take up their equivalents of water, and the glycerine takes up its equivalent; they then distil together. In the receiver, the condensed glycerine, from its greater specific gravity, sinks below the fat acids. Sufficient steam must be supplied, and the temperature regulated, otherwise the elements of glycerine do not take up their equivalents of water, and acroleine is evolved—a body of a very different character. In an ordinary apparatus, the glycerine distilled from neutral fat is not in a sufficiently concentrated state for most purposes; it should, therefore, be concentrated, and if discolered, re-distilled. Thus obtained, it is of sp. gr. 1.240, and contains 94 per cent. of anhydrous glycerine."

This process appears to me, to be much better than any of the preceding, on account of the purity in which the glycerine can be obtained. Distillation might be used to purify the impure product of the old sources.

Glycerine, is a colorless or slightly yellow, transparent liquid, of the consistence of syrup. It is "unctuous to the feel," and has a decidedly sweet taste, with a slightly acrid aftertaste. It is not evaporated by exposure to air, but may be decomposed by a high temperature, when it yields the volatile compound called acroleine ($C^6H^4O^2$,) which is said to attack the eyes and nose in a very painful and even intolerable degree. It may be observed that acroleine, with three parts of water (3HO,) gives the formula for glycerine, which might be called a hydrate of acroleine.

It does not become rancid, nor does it pass spontaneously into a state of fermentation.

It mixes in all proportions with aqueous and alcoholic fluids; also, with vinegar: and, in certain proportions, it mixes with adeps, and fatty matters, and volatile oils.

According to M. A. P. Cap, "it dissolves vegetable acids; all the deliquescent salts; the sulphates of potassa, of soda, and of copper; the nitrates of potassa and of silver; the alkaline chlorides, potassium, sodium, barytes, strontium, bromine, iodine, and even the oxide of lead. Also, it dissolves iodine; iodide of sulphur, potassium, and mercury. It dissolves or suspends the vegetable alkaloids in the same manner as water does; while the products, resulting from its action, may be employed the same as if oil had been the recipient. The salts of morphia dissolve in it completely, even cold, in all proportions. Sulphate of quinine, in the proportion of one-tenth, when it is hot, dissolves in it; but, when cold, separates into clots, which, when triturated with the supernatant liquid, give it the consistence of a cerate, very useful for frictions and embrocations. It is the same with the salts of brucine, strychnine, veratrine, and most preparations of the same order, which enables us to consider that we have now, if not medicinal oils with a vegetable alkaloid base—at least, a series of new preparations which will fulfil a perfectly analagous use in therapeutics."

Dr. Crawcour, of New Orleans, has found it to be an excellent solvent for phosphorus, in the proportion of two grains to the ounce of boiling glycerine; and to be far better of administration than the oil solution, as it is free from nauseous taste, and readily miscible with water.

It might be used for syrup or sugar in many pharmaceutical preparations.

Mr. Wilson speaks highly of it as an agent for the preservation of colors, and has placed specimens of it in the hands of artists for further trial. The same gentleman found it useful as an antiseptic, both for animal flesh and fish; and also proposes it as a preservative of fruit. Its antiseptic effects are evident, from what has been before said of its constitution and properties.

As an external remedy, it is valuable on account of its non-evaporating and unctuous properties, in addition to which it is slightly hygrometric (taking up a small portion of water and retaining it;) thus, both lubricating the surface, and protecting it from the air. According to M. Cap, it penetrates the pores of the skin, and keeps upon its surface, on account of its hygrometric virtues, a sort of permanent moisture. It cicatrizes fissures of the breast, cracks and fissures of the skin, preserving its suppleness and calming the erythism. The same gentleman further states, that Dr. Dallez has pronounced it the most efficacious of all cosmetic agents.

M. Trousseau has used it with benefit in superficial cutaneous diseases; in prurigo; and affections of the ear resulting from irritation of the skin, propagated from the external to the internal auditory apparatus.

M. Velpeau speaks of its use in all cutaneous affections, which would become irritated by fatty or stimulating compounds; and, more especially, in pruriginous affections of the parts of generation, of the anus, or their annexæ.

M. Bazin frequently employs it in eczema, zona, acne, icthyosis, and all cutaneous affections which do not depend on some morbid visceral changes.

Dr. H. A. Johnson, of Chicago, has used it with marked benefit, at the Mercy Hospital, in a case of lepro-psoriasis.

I have used it in treating surfaces, excoriated by frost-bite, with superior benefit. It hastens cicatrization, and forms a sort of artificial cuticle on parts where suppuration has not actually begun.

I have prescribed it in one case of chapped hands, for a man, who declares "there is nothing like it;" have used it, also, in chronic eczema and burns, with very satisfactory effects. It may safely be pronounced one of the best of external remedies.

Being hydro-carbonaceous in its constitution, and somewhat analagous to cod-liver oil, it would seem to be well adapted to consumptive and scrofulous diseases: a supposition which facts, so far as its use has yet gone, strongly corroborate. Whether its effects, in these cases, are owing to its being used by the system as respiratory food, and so preventing the oxygenation of the tissues; or, rather, to its favoring a healthy digestion, and assisting in the assimilation of nutritious ingesta; has not been determined. However, the latter hypothesis seems to be the more tenable from the nature of the case; besides, observation goes far to sustain it. Dr. Crawcour, (the only person known to have used it internally, before its introduction into the hospital,) who has witnessed its effects in these diseases, asserts that "it, in a short period, relieves the cough, improves the digestive powers, and appears to increase the fat-producing principle in phthisical patients." Its *modus operandi* has not yet been determined; but, from what we already know of its miscible and penetrating qualities, we infer that it readily passes into the circulation, in which, and in its course through the liver, it meets with lactic and other organic and fatty acids, which are capable of being modified, so as to combine with it as a fatty base: so that we have, present in the blood, the elements necessary for the deposition of adipose matter. According to this hypothesis, it would be especially useful in *tabes mesenterica*, and where there is impairment of the digestion, from deficient and unhealthy secretion of bile and pancreatic juice; or, where there exists lesions of the lacteal system, preventing the absorption of chyle, which is generally considered to be an emulsion of the oleaginous ingesta.

In its internal use, it has great advantage over cod-liver oil, on account of its blandness, and favoring instead of destroying the digestion, and nauseating the patient. In children, it is easy of administration, on account of its sweet and agreeable taste.

In the latter part of October, 1855, by permission of Dr. N. S. Davis, I used it at the Mercy Hospital, in the proportion of four parts to one of the syr. of the iodide of iron, in the case of a man about 40 years of age, who had been using the cod-liver oil, but was obliged to discontinue it, on account of its nauseating and irritating effects on the stomach. This man, at the time, was much emaciated, much troubled with coughing, and expectorating daily nearly a pint of thick, tenacious mucus, freely mixed with tubercular matter in a granular form. The infra-clavicular space was considerably fallen in, and all the symptoms and signs plainly evinced quite an advanced stage of the disease. He had been under treatment for several weeks, just previous, for chronic diarrhœa, which was not entirely cured.

In about a week after commencing the use of this prescription, the cough and expectoration ceased, and the patient was much improved generally. He has continued its use up to the present time, Jan. 10, 1856; has regained his natural fulness and rotundity, and is fast regaining his strength.

Dr. Davis subsequently used the same remedy in a case of incipient tuberculosis, with equally satisfactory results. Dr. De Laskie Miller recently prescribed it in a more advanced stage of the disease, with the most happy effects. In conclusion, I believe it a most valuable addition to the list of curative agents.

